# Intraoperative monitoring parameters and postoperative delirium: Results of a prospective cross-sectional trial

**DOI:** 10.1097/MD.0000000000024160

**Published:** 2021-01-08

**Authors:** Carolin Jung, Lukas Hinken, Moritz Fischer-Kumbruch, Dominik Trübenbach, Rieke Fielbrand, Isabel Schenk, Oliver Diegmann, Terence Krauß, Dirk Scheinichen, Barbara Schultz

**Affiliations:** Department of Anaesthesiology and Intensive Care Medicine, Hannover Medical School, Hannover, Germany.

**Keywords:** EEG monitoring, mean arterial pressure, minimum alveolar concentration, postoperative delirium, sevoflurane

## Abstract

Postoperative delirium (PODE) can be associated with severe clinical complications; therefore, preventive measures are important. The objective of this trial was to elucidate whether haemodynamic or electroencephalographic (EEG) monitoring parameters during general anaesthesia or sevoflurane dosage correlate with the incidence of PODE. In addition, sevoflurane dosages and EEG stages during the steady state of anaesthesia were analyzed in patients of different ages.

Eighty adult patients undergoing elective abdominal surgery received anaesthesia with sevoflurane and sufentanil according to the clinical routine. Anaesthesiologists were blinded to the EEG. Haemodynamic parameters, EEG parameters, sevoflurane dosage, and occurrence of PODE were analyzed.

Thirteen patients (4 out of 33 women, 9 out of 47 men) developed PODE. Patients with PODE had a greater mean arterial pressure (MAP) variance (267.26 (139.40) vs 192.56 (99.64) mmHg^2^, *P* = .04), had a longer duration of EEG burst suppression or suppression (27.09 (45.32) vs 5.23 (10.80) minutes, *P* = .03), and received higher minimum alveolar sevoflurane concentrations (MAC) (1.22 (0.22) vs 1.09 (0.17), *P* = .03) than patients without PODE. MAC values were associated with wide ranges of EEG index values representing different levels of hypnosis.

The results suggest that, in order to prevent PODE, a great variance of MAP, higher doses of sevoflurane, and deep levels of anaesthesia should be avoided. Titrating sevoflurane according to end-tidal gas monitoring and vital signs can lead to unnecessarily deep or light hypnosis. Intraoperative EEG monitoring may help to prevent PODE.

## 1 Introduction

Delirium is defined as a disturbance in attention, awareness, and cognition that develops over a short period of time and fluctuates in severity.^[1]^ For the most part, delirium is a transient neuropsychiatric disorder. However, patients affected by delirium show a greater incidence of postoperative complications and have increased rates of adverse outcome such as poor functional outcome, postoperative cognitive decline, higher mortality, greater medical expenses, and longer hospitalisation.^[2–5]^

Postoperative delirium (PODE) is a complication that occurs in patients of all ages, but it appears to be especially frequent in elderly and multimorbid patients.^[6,7]^ According to Dasgupta and Dumbrell, its incidence after non-cardiac surgery varies between 5% and more than 50%.^[8]^ Scholz and colleagues found a median PODE rate of 23.9% in 11 studies included in a meta-analysis of risk factors for postoperative delirium among older patients undergoing gastrointestinal surgery.^[9]^ PODE is a complex multifactorial syndrome and a substantial amount of PODE seems to be due to unmodifiable risk factors like inflammation and comorbid disease.^[10]^ Modifiable risk factors have also been identified, such as disturbances in intraoperative homeostasis, benzodiazepines, anticholinergic medication, inadequately controlled pain, and excessive anaesthesia depth.^[10–12]^

Among intraoperative monitoring parameters, haemodynamic parameters, such as blood pressure and heart rate, have been reported to differ between PODE-positive and PODE-negative patients.^[13,14]^

Processed electroencephalography (EEG) is a method of neuromonitoring for estimating anaesthetic depth by analysing EEG waveforms. One argument for its use is that it avoids very light stages of anaesthesia.^[15]^ In recent years, several authors have reported that a higher rate of EEG stages typical for deep anaesthesia is associated with an increased incidence of PODE.^[16]^

EEG monitoring for the prevention of delirium is recommended by the European Society of Anaesthesiology (ESA), and it is suggested by other organisations.^[7,17]^ Nevertheless, there are authors who take the view that current evidence is insufficient to recommend the use of EEG monitoring to reduce the risk of postoperative delirium.^[18]^

The objective of the analysis presented in this paper is to elucidate whether EEG or haemodynamic monitoring parameters during general anaesthesia or sevoflurane dosage correlate with the incidence of postoperative delirium. In addition, the relationship between sevoflurane dosages and EEG stages during the steady state of anaesthesia is analyzed.

The data set was derived from a prospective single-centre cross-sectional study and comprises courses of anaesthesia performed with sevoflurane for maintenance of anaesthesia. The analysis is intended to provide information on how to improve the quality of general anaesthesia, especially for elderly people.

## 2 Methods

The single-centre, prospective, cross-sectional study was conducted at a large academic hospital with 1520 beds. The responsible ethics committee's approval was issued on February 12, 2016 (Ethics Committee of Hannover Medical School, Hannover, Germany, Approval No. 3070-2016).

### 2.1 Patient inclusion and exclusion

Patients were eligible for the study if they were older than 18 years and if they had to undergo planned major abdominal surgery. Exclusion criteria were incapability to consent, insufficient command of the German language, severe hypoacusis or anacusis, and revision surgery during the investigation period.

Between April 2016 and September 2017, a total of 99 patients aged 42 to 84 years participated in the study; 40 were female and 59 were male. Patients receiving continuous IV propofol (n = 5) or a combination of sevoflurane and IV propofol (n = 2) for maintenance of anaesthesia were excluded from the analysis.^[19–22]^ Patients with prolonged postoperative sedation at the intensive care unit (ICU) (n = 9) were also excluded since proper screening for delirium was not feasible. Two patients’ data was not used for the analysis as they withdrew their consent to participate in the study, and 1 patient had to be excluded due to missing data. The data analysis regarding intraoperative monitoring included 80 patients receiving balanced anaesthesia with sevoflurane and sufentanil.

### 2.2 Preoperative assessment

The day before surgery, the Montreal Cognitive Assessment (MoCA) was used to assess the patient's preoperative cognitive function. The MoCA is an instrument developed to detect mild cognitive impairment. It tests short-term memory, delayed recall, visuospatial abilities, executive functions, language abilities, attention, concentration, calculation, and orientation to time and place.^[23]^ The patients were also screened for major depression as a differential diagnosis of PODE using the Patient Health Questionnaire-2 (PHQ-2).^[24,25]^

The patients’ physical status was recorded using the American Society of Anesthesiologists (ASA) physical status classification system.^[26]^

Comorbidities were classified by means of the Charlson Comorbidity Index (CCI), a weighted index that takes into account the number and the seriousness of comorbid disease.^[27,28]^

### 2.3 Anaesthesia

The responsible anaesthesiologists were asked to perform anaesthesia according to clinical routine. If the patient asked for oral premedication, it was administered about 1 hour before induction of anaesthesia. The patients were given 3.75 mg or 7.5 mg midazolam; one patient received 150 μg clonidine. Hypnotics used for induction were propofol or thiopental, depending on patient characteristics and the anaesthesiologist's clinical choice. Further anaesthetics administered during induction were sufentanil and either atracurium or rocuronium. Tracheal intubation was performed after administration of the muscle relaxant. Maintenance of anaesthesia was performed with inhaled sevoflurane and intravenous boli of sufentanil. Normoventilation was intended. Depth of anaesthesia was maintained according to clinical assessment. The responsible anaesthesiologists and nurses were blinded to the EEG. After surgery, the patients were transferred to the ICU and kept sedated until they reached a stable condition.

### 2.4 Intraoperative data

During anaesthesia, the following parameters were recorded: ventilation parameters including end-tidal sevoflurane concentration, electrocardiography (ECG), pulse oximetry, capnography, non-invasive blood pressure, arterial blood pressure, and temperature. Additionally, the EEG (Narcotrend-Compact M, MT Monitor Technik GmbH & Co. KG, Bad Bramstedt, Germany) was used to document depth of hypnosis. The Narcotrend-Compact M indicates the raw EEG and the Narcotrend-Index (NI), where an index of 0 defines a completely suppressed EEG corresponding to very deep hypnosis and 95 to 100 to an awake patient. The index values 0 to 100 correspond to the Narcotrend stages F to A.

For this analysis, end-tidal sevoflurane concentrations, blood pressure values, and NI values collected at 1-minute intervals were used.

### 2.5 Postoperative delirium testing

For measuring postoperative delirium, the 3D-CAM was selected, a 3-minute structured diagnostic assessment using the CAM-algorithm.^[29]^ The 3D-CAM comprises the features a) acute change and fluctuating course, b) inattention, c) disorganized thinking, and d) altered level of consciousness. The patient was classified as delirious if features a) and b) plus either feature c) or d) were present.^[29]^ Patients were examined with regard to signs of delirium twice daily on postoperative days 1 to 7. The examinations were performed by members of the study group.

### 2.6 Parameters calculated for statistical analysis

Duration of anaesthesia was calculated as the time between start of induction and the end of the last suture. For this time period, the duration of Narcotrend Index in stages F_0_/F_1_, sevoflurane and sufentanil dosages, as well as different blood pressure parameters, were determined.

Narcotrend stage F_0_ is characterised by the burst suppression pattern, showing alternating bursts of EEG activity and periods with a very flat EEG curve. From F_0_ to F_1_, the proportion of the very flat periods becomes greater until a complete suppression is reached.

The median NI was calculated to provide a measure of the NI level during the steady state of anaesthesia. The median NI was calculated for the time period between end of induction of anaesthesia and last suture.

The age-adjusted minimum alveolar concentrations (MAC) of sevoflurane were calculated using the sevoflurane in O_2_ dosages provided in the Summary of Product Characteristics.^[30]^

### 2.7 Statistical analysis

Statistical analysis was performed using logistic regression, analysis of regression and correlation as well as chi square test and Fisher's exact test. Statistical significance was assumed at *P* < .05.

Logistic regression was used using occurrence of PODE as dependent variable. The relevant covariates were analyzed using univariate logistic regression. Due to the limited sample size, applying a multifactorial model including all relevant covariates was not deemed sensible.^[31]^

The statistical analysis was performed with the statistical software SAS (SAS Institute, Cary, USA), version 9.3. Cases where some data was missing were only excluded for analyses involving the missing data.

## 3 Results

### 3.1 Patients

Of the 80 patients included in the analysis, 13 developed PODE, while 19 patients of the basic data set comprising 99 patients developed PODE.

With the exception of one patient, who received a hemi-hepatectomy due to echinococcosis, all patients received surgery due to a diagnosed or suspected malignant disease. The surgical procedures performed in our population included surgery in patients with gynaecological malignancies (n = 3), liver surgery (n = 39), pancreatic surgery (n = 16), gastric surgery (n = 3), oesophageal surgery (n = 8), intestinal surgery (n = 4), and other surgery (n = 7).

The mean age among delirium-positive patients was 70.68 (9.41) years. For delirium-negative patients, a mean age of 65.46 (10.61) years was calculated (*P* = .11). In 4 of 33 (12%) female patients and 9 of 47 (19%) male patients, a PODE was diagnosed (*P* = .54).

### 3.2 Preoperative assessment

The 80 patients included had a high burden of preoperative comorbidities. All participating patients had a Charlson Comorbidity Index (CCI) >2. The CCI ranged from 3 to 18. There was no statistically significant difference regarding CCI between patients developing delirium and those that did not (9.46 (3.76) vs 9.00 (3.19), mean (SD), *P* = .64). Among the 13 delirium-positive patients, the American Society of Anesthesiologists (ASA) classification was distributed as follows: class I: 0 patients (0.00%); class II: 7 patients (53.85%); class III: 6 patients (46.15%). Out of the 67 patients without delirium, 1 patient (1.49%) was in class I, 36 (53.73%) were in class II, and 30 (44.78%) in class III. There was no significant difference in American Society of Anesthesiologists (ASA) classification between the groups (*P* = 1.0).

For the MoCA test and the PHQ-2 test, there was no significant difference between delirium-positive and delirium-negative patients (MoCA: 24.83 (2.29) vs 24.97 (3.25), mean (SD), *P* = .89; PHQ-2: 1.46 (2.54) vs 1.46 (1.61), mean (SD), *P* = 1.0).

### 3.3 Premedication

Table 1 shows the number of patients with and without oral midazolam premedication in the delirium-positive and in the delirium-negative group. There was no significant difference in PODE incidence between patients with and without oral midazolam premedication (*P* = .12); therefore, oral midazolam premedication was not considered a confounder in this analysis.

**Table 1:** Number of patients without premedication and with oral midazolam premedication for the delirium-positive and delirium-negative group.

	Delirium-positive	Delirium-negative
No oral premedication	3	4
Midazolam 3.75 mg	6	27
Midazolam 7.5 mg	4	35

One delirium-negative patient received 150 μg oral clonidine.

### 3.4 Intraoperative parameters

Intraoperative parameters of the patient groups with and without PODE are shown in Table 2. For technical reasons, for 2 patients no blood pressure values were available, for 1 patient no sevoflurane concentrations, and for 2 patients no EEG data. The mean length of anaesthesia was 234.59 (59.38) minutes for delirium-positive and 212.05 (79.21) minutes for delirium-negative patients (mean (SD), *P* = .35).

**Table 2:** Delirium-positive and delirium-negative patients, intraoperative parameters.

	Delirium-positive		Delirium-negative		*P*
Duration of anaesthesia (min)	234.59	(59.38)	212.05	(79.21)	.35
Duration of EEG stage F_0_/F_1_ (min)	27.09	(45.32)	5.23	(10.80)	.03
Sevoflurane vol.% end-tidal	1.75	(0.30)	1.64	(0.27)	.28
MAC sevoflurane, age-adjusted	1.22	(0.22)	1.09	(0.17)	.03
Sevoflurane >2.0 vol.% end-tidal (min)	54.42	(68.39)	25.76	(44.76)	.08
Sufentanil (μg kg^−1^ h^−1^)	0.41	(0.14)	0.49	(0.20)	.18
MAP <60 mmHg (min)	6.58	(5.53)	8.58	(13.12)	.61
MAP <70% of the baseline value (min)	2.64	(2.42)	3.03	(3.63)	.73
Variance MAP (mmHg^2^)	267.26	(139.40)	192.56	(99.64)	.04

Data are mean (standard deviation). EEG = electroencephalogram, MAC = minimum alveolar concentration, MAP = mean arterial pressure.

#### 3.4.1 MAP

**Figure 1:**
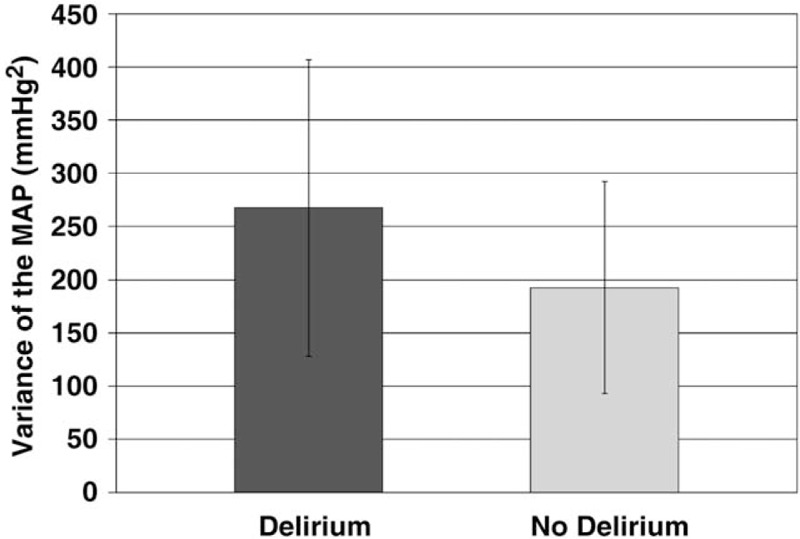
Intraoperative variance of mean arterial pressure in patients with and without postoperative delirium. MAP = mean arterial pressure.

The variance of intraoperative MAP showed a significant difference, with a noticeably higher variance for delirium-positive patients (*P* = .04) (Fig. 1, Table 2). The duration of MAP <60 mmHg was not significantly different in patients with and without PODE (*P* = .61) (Table 2). One patient had a phase of 19 minutes with MAP <60 mmHg, but did not develop signs of delirium. For the duration of MAP <70% of baseline, there was no significant difference, either (*P* *=* .73). The longest period with MAP <70% of baseline was 17 minutes. In this case, the mean MAP was 64.7 mmHg, and the patient did not develop PODE.

#### 3.4.2 Sevoflurane and sufentanil administration

**Figure 2:**
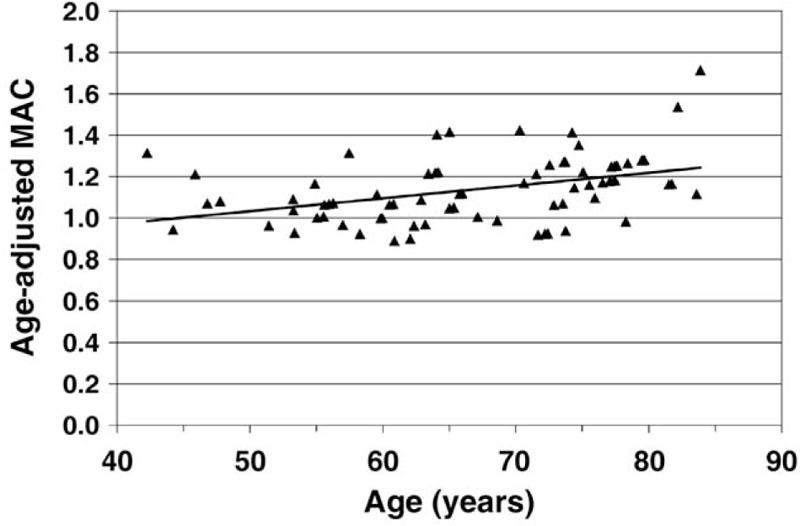
Mean age-adjusted MAC values of sevoflurane plotted against patient age. MAC = minimum alveolar concentration.

Patients with PODE received a higher mean sevoflurane dose than patients without PODE (MAC 1.22 (0.22) vs MAC 1.09 (0.17)) (*P* = .03) (Table 2). Older patients displayed higher MAC values than younger patients (*P* < .001). For example, as shown in Figure 2, the 2 patients with the highest MAC values were over 80 years old. The mean duration of sevoflurane administration with an end-tidal concentration greater than 2.0 vol.% was longer for patients with PODE (*P* = .08). The difference was not significant (Table 2).

The mean dose of sufentanil per kilogram of body weight and hour was less for patients with PODE than for patients without PODE. However, these results were not significantly different (*P* = .18) (Table 2).

#### 3.4.3 Intraoperative EEG

**Figure 3:**
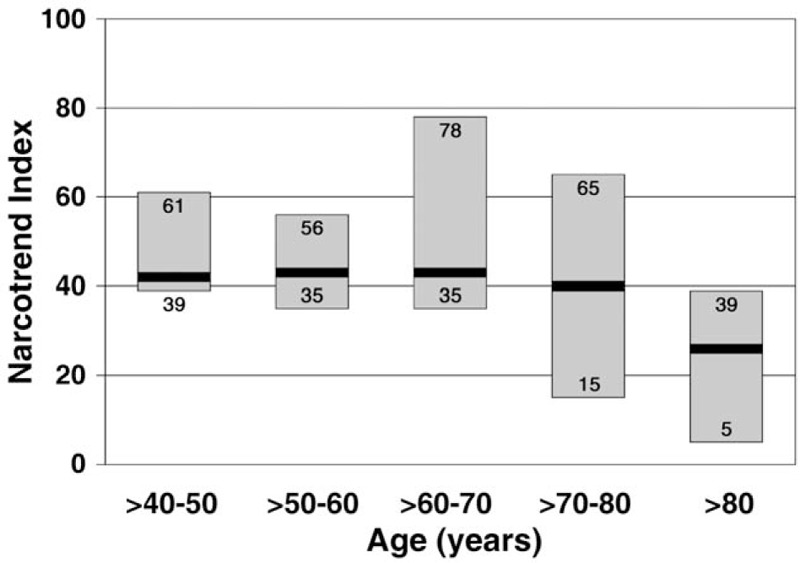
Range of the median Narcotrend Index at different patient ages. The bold black lines within each bar represent the medians of the ranges.

The mean duration of the burst suppression or suppression stage was significantly longer in patients with PODE (*P* = .03) (Table 2). On average, older patients reached deeper levels of hypnosis (low NI); the regression coefficient (NI and age) was significant (b = −0.3581, *P* = .003). Figure 3 shows the ranges of the median NI for patients of different ages.

**Figure 4:**
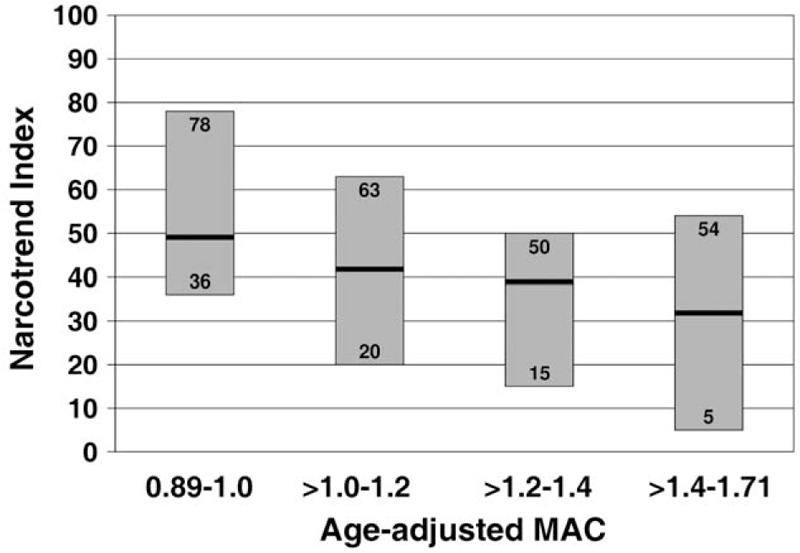
Range of the median Narcotrend Index at different MAC values of sevoflurane. The bold black lines within each bar represent the medians of the ranges. MAC = minimum alveolar concentration.

**Figure 5:**
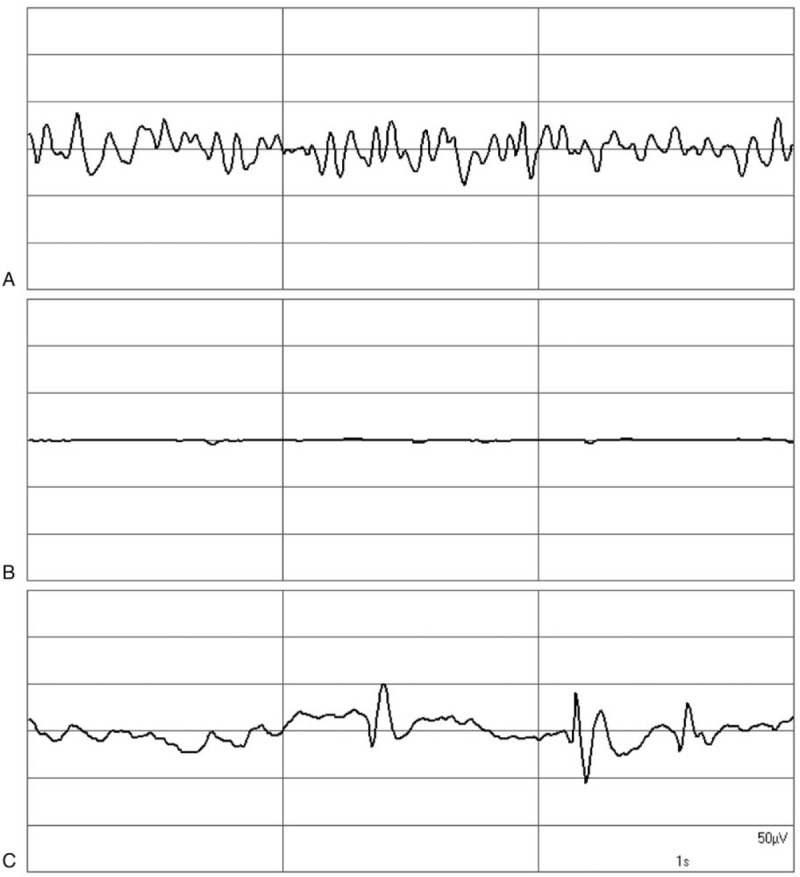
EEG graphs during the steady state of anaesthesia as observed in different patients. (A) EEG at NI of 79, (B) suppression EEG, (C) epileptiform discharges. EEG = electroencephalogram.

For an evaluation of NI and age-adjusted MAC values, the MAC values were divided into 4 groups. Figure 4 shows the range of the median NI in the 4 groups. The highest NI values were observed in the group of the lowest MAC values (>0.89–1.0), whereas the lowest NI values occurred in the group with the highest MAC values (>1.4–1.71). All 4 MAC groups show a great variability of NI. Visual screening of the raw EEGs revealed a wide variation of patterns during the steady state. Figure 5 shows 3 examples: an EEG consisting mainly of waves with higher frequencies indicating a light level of hypnosis (Fig. 5A), a completely suppressed EEG from a very deep stage of hypnosis (Fig. 5B), and an EEG with epileptiform potentials (Fig. 5C). Due to prolonged postoperative ventilation, the patient with the epileptiform potentials was excluded from this analysis.

## 4 Discussion

The objective of this analysis was to evaluate whether haemodynamic parameters, EEG parameters or sevoflurane dosage correlate with the incidence of PODE after balanced anaesthesia with sevoflurane and sufentanil. The comparison between patients with PODE and without PODE makes it evident that patients with PODE displayed a greater variability of MAP, higher MAC values, and a longer duration of burst suppression or suppression EEG. Furthermore, as shown in Figure 4, MAC values were associated with wide ranges of median NI values representing different levels of anaesthesia. The incidence of PODE in this analysis was 16.25%. Rates of PODE in patients receiving abdominal surgery, ranging from hernia repair and appendectomies to more invasive procedures, are known to vary between 17% to 51%.^[32]^

### 4.1 Intraoperative MAP

Intraoperative MAP variance was significantly increased in patients with PODE. For the parameters “MAP <60 mmHg” and “MAP <70% of baseline”, the difference between the 2 groups was not significant. Similar observations regarding intraoperative hypotension and blood pressure fluctuations and their impact on early PODE after non-cardiac surgery were published by Hirsch and co-workers.^[33]^ In our study, sufentanil was administered via bolus. In 2017, the European Society of Anaesthesiology (ESA) published an evidence-based and consensus-based guideline on PODE.^[7]^ In the ESA guideline, it is suggested to use a continuous intraoperative analgesia regimen (e.g., with remifentanil).^[7]^ It can be expected that the variance of MAP is reduced in case of a continuous intraoperative administration of analgesics.

### 4.2 MAC of sevoflurane

In the current analysis, patients with PODE were, on average, older than patients without PODE, but statistical significance was not reached. Patients with PODE had significantly higher MAC values. With incremental age, MAC values showed a significant increase. A possible explanation could be that the attending anaesthesiologists titrate depth of anaesthesia according to end-tidal concentration of sevoflurane instead of using MAC values which include an age adjustment.

There was high interindividual variability of the median NI values at defined MAC values in the steady state of anaesthesia. For example, for >1.0 to 1.2 MAC, the highest median NI value of 63 (D_0_) indicated a light to moderate EEG stage, while the deepest value of 20 (E_2_) was at the border to the burst suppression state. In this study, the anaesthesiologists were blinded to the EEG. Using the EEG as a measure for guiding anaesthesia could have avoided extremely light and extremely deep courses of anaesthesia.

### 4.3 Depth of anaesthesia monitoring

In our analysis, the mean duration of burst suppression or suppression stage was significantly higher among patients with PODE than in patients without PODE. The ESA guideline on PODE states that intraoperative neuromonitoring is important in order to avoid unnecessarily deep anaesthesia, often reaching burst suppression in elderly patients. The guideline recommends monitoring depth of anaesthesia (grade A, strong recommendation).^[7]^ In 2018, MacKenzie and co-workers performed a systematic review and meta-analysis which examined the relationship between processed EEG monitoring and PODE and cognitive dysfunction. They concluded that processed EEG-guided anaesthesia was associated with a decrease in postoperative delirium.^[34]^ In a Cochrane analysis from 2018, Punjasawadwong and co-workers came to a similar conclusion.^[35]^ Lima and colleagues (2018) reported that the implementation of guidelines with predefined targets for haemodynamic and depth of anaesthesia monitoring was associated with a significant improvement in postoperative outcome in patients undergoing major open abdominal surgery for cancer. After the implementation, the rate of patients with PODE dropped from previously 16% to 8% (*P* = .005).^[36]^

Many different risk factors have been reported in literature as causative or promoting factors for the development of PODE.^[7–9]^ The results of our analysis support the observations that very deep levels of sevoflurane anaesthesia and pronounced blood pressure variations contribute to the occurrence of PODE.

### 4.4 Limitations

The data set that was used for this analysis has some limitations. The sample consists of 80 patients - one reason for this was that the data collection was a very time-consuming task; some patients, e.g., with prolonged sedation and ventilation at the ICU, were excluded. Due to the sample size, multivariate logistic regression was not reasonable in the delirium analysis. Nevertheless, the number of EEGs recorded in the included patients was sufficient to show a wide variation of patterns during the steady state.

## 5 Conclusions

In conclusion, the results of this data analysis suggest that, in order to prevent PODE, higher doses of sevoflurane, which could lead to very deep levels of hypnosis, and great variance of MAP should be avoided. Titrating sevoflurane according to end-tidal gas monitoring and vital signs can lead to unnecessarily deep or light hypnosis. The implementation of EEG monitoring as a standard may help to prevent PODE.

## Acknowledgments

We thank PD Dr. Ulrich Grouven for advice regarding statistics.

We acknowledge support by the German Research Foundation (DFG) and the Open Access Publication Fund of Hannover Medical School (MHH).

## Author contributions

**Conceptualization:** Carolin Jung, Lukas Hinken, Moritz Fischer-Kumbruch, Dominik Trübenbach, Terence Krauß, Dirk Scheinichen, Barbara Schultz.

**Data curation:** Carolin Jung, Lukas Hinken, Barbara Schultz.

**Formal analysis:** Carolin Jung, Lukas Hinken, Barbara Schultz.

**Investigation:** Carolin Jung, Lukas Hinken, Moritz Fischer-Kumbruch, Dominik Trübenbach, Rieke Fielbrand, Isabel Schenk, Oliver Diegmann, Dirk Scheinichen.

**Methodology:** Carolin Jung, Lukas Hinken, Moritz Fischer-Kumbruch, Dominik Trübenbach, Terence Krauß, Dirk Scheinichen, Barbara Schultz.

**Project administration:** Carolin Jung, Lukas Hinken, Moritz Fischer-Kumbruch, Dominik Trübenbach, Dirk Scheinichen.

**Supervision:** Carolin Jung, Lukas Hinken, Dirk Scheinichen.

**Validation:** Carolin Jung, Lukas Hinken, Moritz Fischer-Kumbruch, Dominik Trübenbach, Rieke Fielbrand, Isabel Schenk, Oliver Diegmann, Terence Krauß, Dirk Scheinichen, Barbara Schultz.

**Visualization:** Lukas Hinken, Dominik Trübenbach, Barbara Schultz.

**Writing – original draft:** Carolin Jung, Lukas Hinken, Barbara Schultz.

**Writing – review & editing:** Carolin Jung, Lukas Hinken, Moritz Fischer-Kumbruch, Dominik Trübenbach, Rieke Fielbrand, Isabel Schenk, Oliver Diegmann, Terence Krauß, Dirk Scheinichen, Barbara Schultz.

## Correction

The unit for the y axis on Figure 1 originally appeared incorrectly as mmHg and has been corrected to mmHg^2^.
